# Supplementary material for: Stacked binding of a PET ligand to Alzheimer’s tau paired helical filaments
Source: Nat Commun. 2023 May 26;14:3048. doi: 10.1038/s41467-023-38537-y (PMC10220082; doi:10.1038/s41467-023-38537-y)
Supplement: Supplementary file 1 — Supplementary Information [file 41467_2023_38537_MOESM1_ESM.pdf]

Supplementary Information for

Stacked binding of a PET ligand to Alzheimer's tau paired helical filaments

Gregory E. Merz, Matthew J. Chalkley, Sophia Tan, Eric Tse, Joanne Lee, Stanley B. Prusiner, Nick A. Paras, William F. DeGrado, and Daniel R. Southworth

Correspondence to: Daniel Southworth, daniel.southworth@ucsf.edu

This PDF file includes:

Supplementary Figures 1 to 10
Supplementary Tables 1 to 3

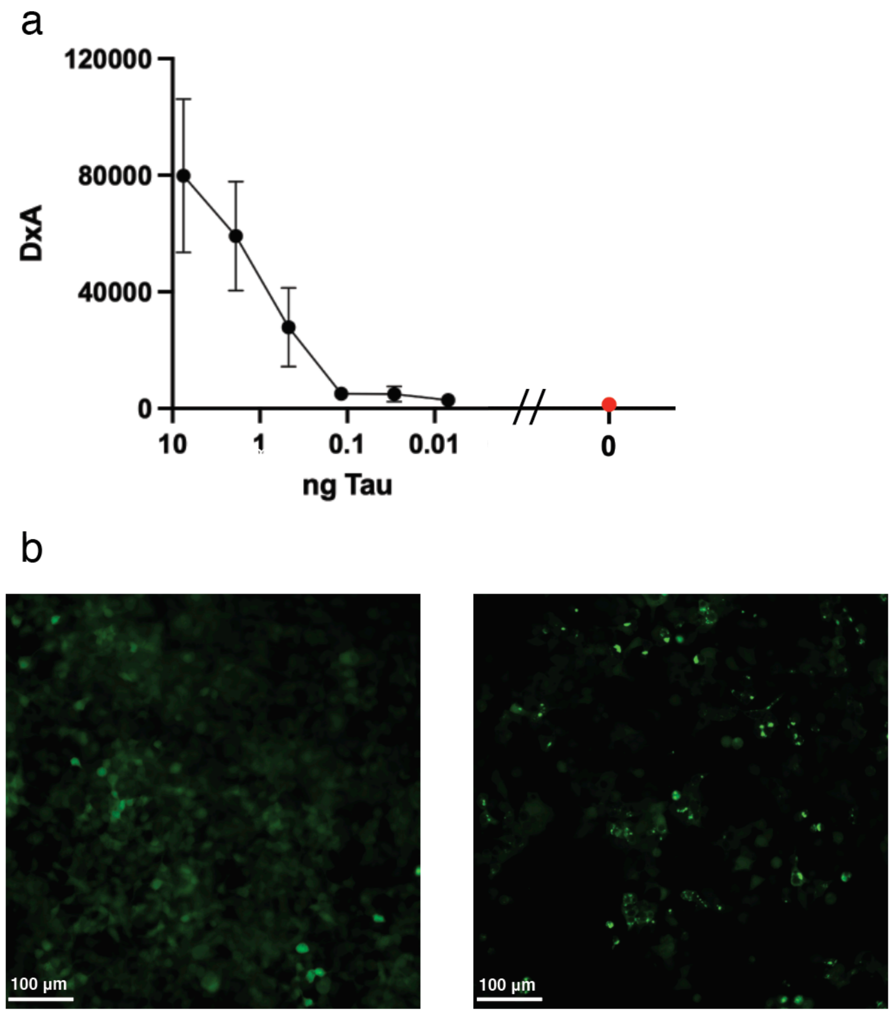

Supplementary Figure 1. Infectivity of tau filaments purified from human AD brain tissue for cryo-EM structure determination.

Infectivity of the partially purified tissue from which the GTP-1 co-structure was solved, as measured by a cell-based fluorescence assay. The tissue was incubated for 3 days with HEK-293T cells that express the repeat domain of 4R tau–containing mutations P301L and V337M fused to YFP[1]. The level of infectivity was measured by the size and brightness of fluorescent puncta formed in the cells, which is quantified as DxA (see Methods). **a.** Quantification of partially purified tissue infectivity over a range of total tau in the sample, as determined using a total tau homogenous time-resolved fluorescence (HTRF) assay [2]. As increasing amounts of tau were added to the cells, the DxA increased, indicating that our imaged sample was pathogenic and disease relevant. The red point represents a control with no tau added. Data are presented as mean values +/- SD, using n=4 biologically independent samples. **b.** Representative images from a control with no tau (left) and a sample incubated with 7.5 ng total tau (right). The diffuse fluorescence in the left image indicates a lack of infectivity, while the distinct puncta in the right image are characteristic of pathogenicity.

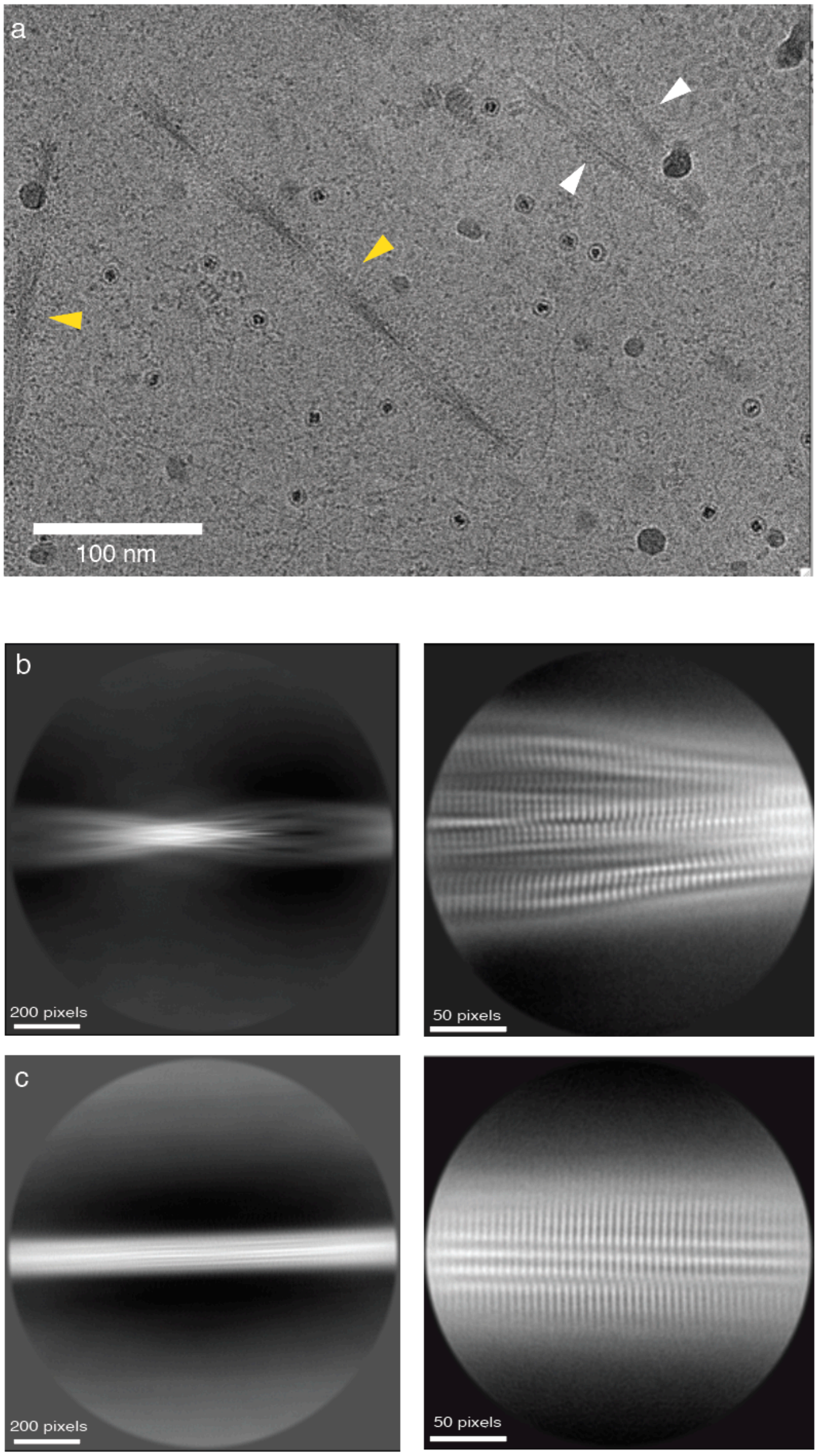

Supplementary Figure 2. Micrograph and 2D class averages of AD filaments used for 3D structure determination.

a. Single representative cryo–electron micrograph (out of 15,160) showing both paired helical filaments (PHFs) and straight filaments (SFs) purified from AD patient tissue. PHF (gold arrows) and SF (white arrows) determination is based on crossover distance and comparisons to previous

image data[3]. Representative reference-free 2D class averages are shown for (**b**) PHFs and (**c**) SFs, with box sizes 1,200 pixels downscaled to 300 pixels (left) and 288 pixels without downscaling (right).

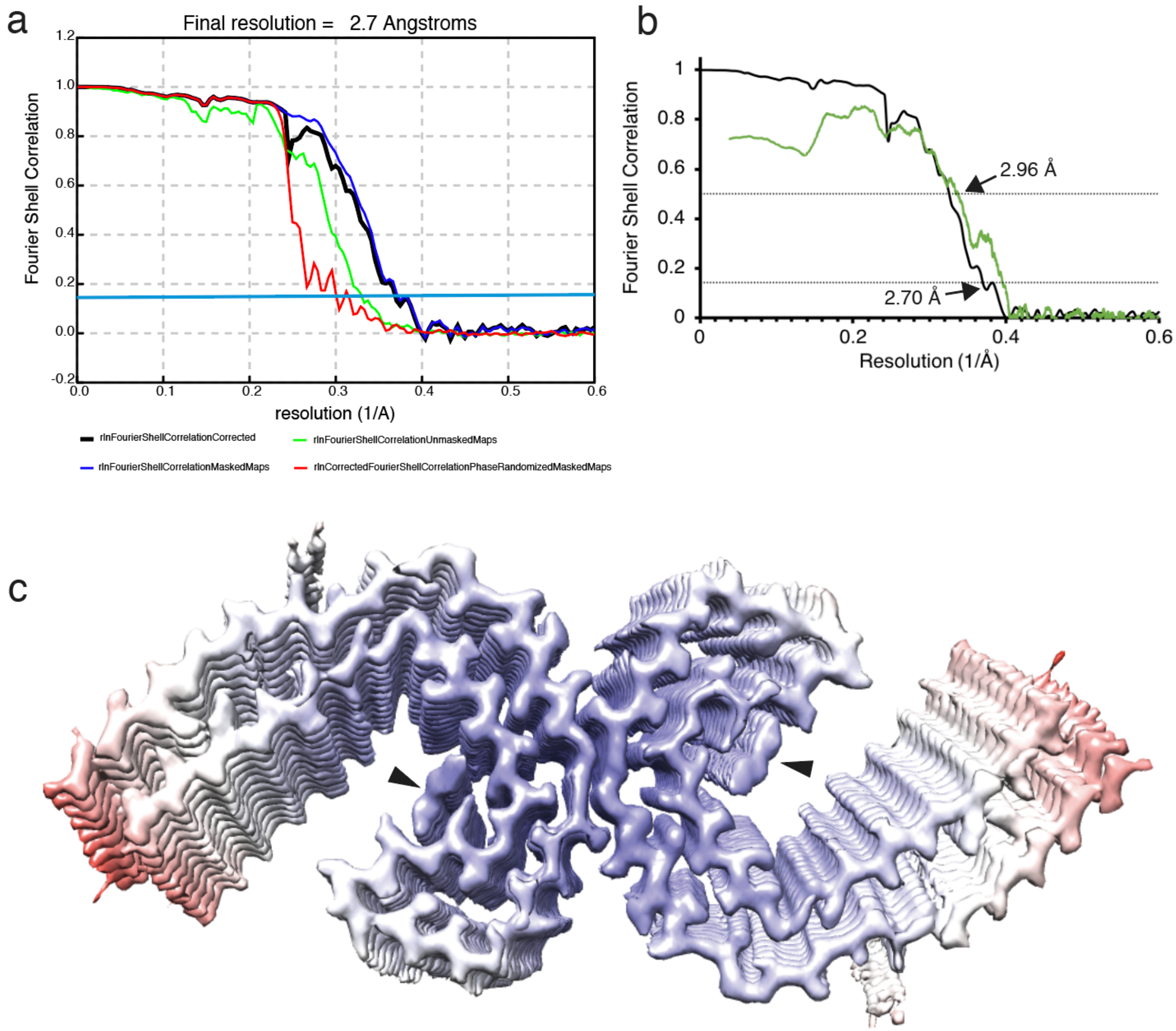

Supplementary Figure 3. Fourier shell correlation (FSC) curves and local resolution map. **a.** FSC curves for two independently refined cryo-EM half maps of the tau PHF:GTP-1 structure. **b.** Corrected FSC curve from (**a**) in black and the FSC curve for the refined atomic model against the final cryo-EM map in green. **c**, Local resolution map of tau PHF:GTP-1 showing high resolution (~2.5 Å) of the filament core and GTP-1 ligand (black triangle).

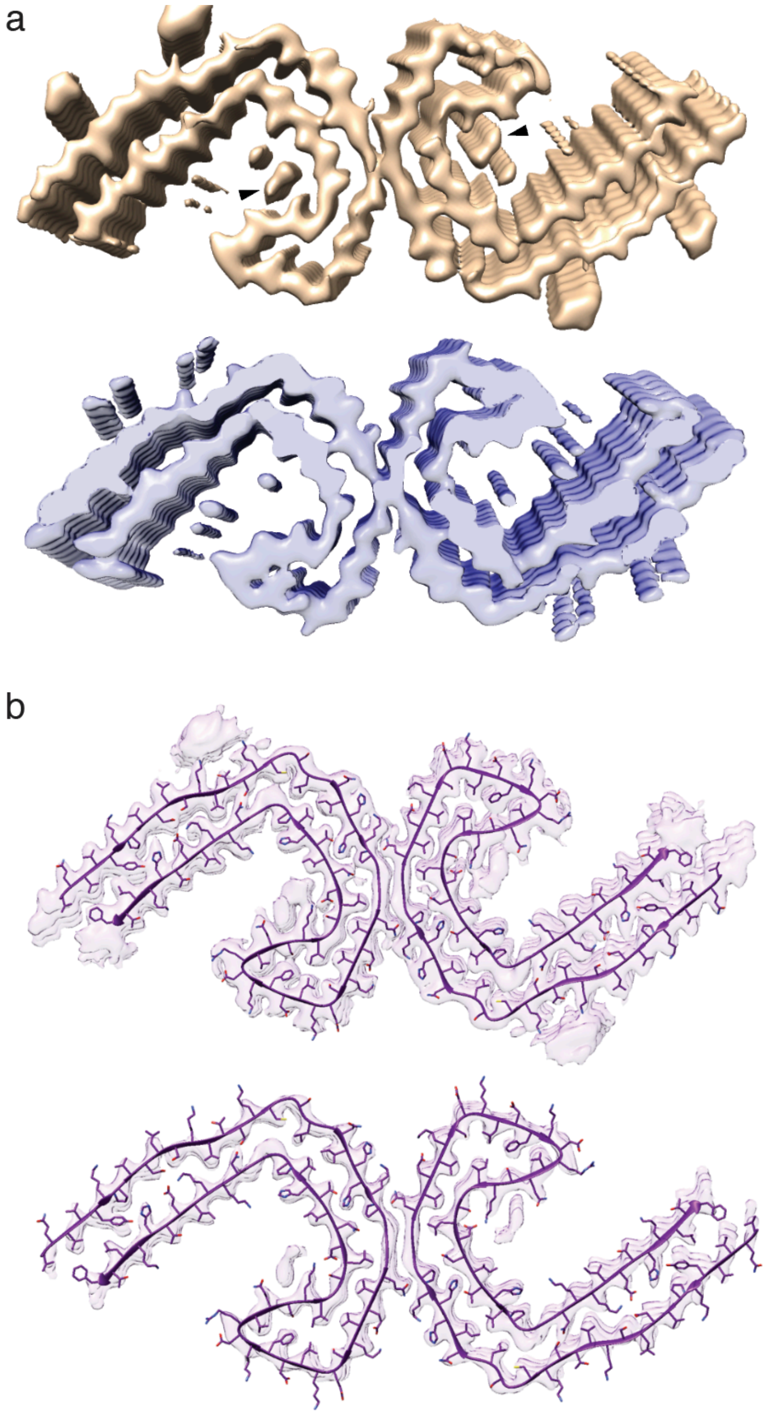

Supplementary Figure 4. PHF:GTP-1 maps indicate high specificity and occupancy for GTP-1.

a. Cryo-EM maps of tau PHF:GTP-1 (top: gold), in comparison to the previously solved structure (EMD-0259 [https://www.ebi.ac.uk/emdb/EMD-0259]; bottom: blue) low-pass filtered to 5 Å. Additional density ascribed to GTP-1 is indicated (arrows). No other densities unique to the PHF:GTP-1 map are identified, indicating specific binding. **b.** Cryo-EM map of AD PHFs in complex with GTP-1 low-pass filtered to 3.5 Å at Chimera threshold level 0.0095 (top, gold, σ =

3.0) and 0.0243 (bottom, blue). While other densities surrounding the amyloid filament disappear at high threshold, the density corresponding to GTP-1 remains, indicating high binding occupancy.

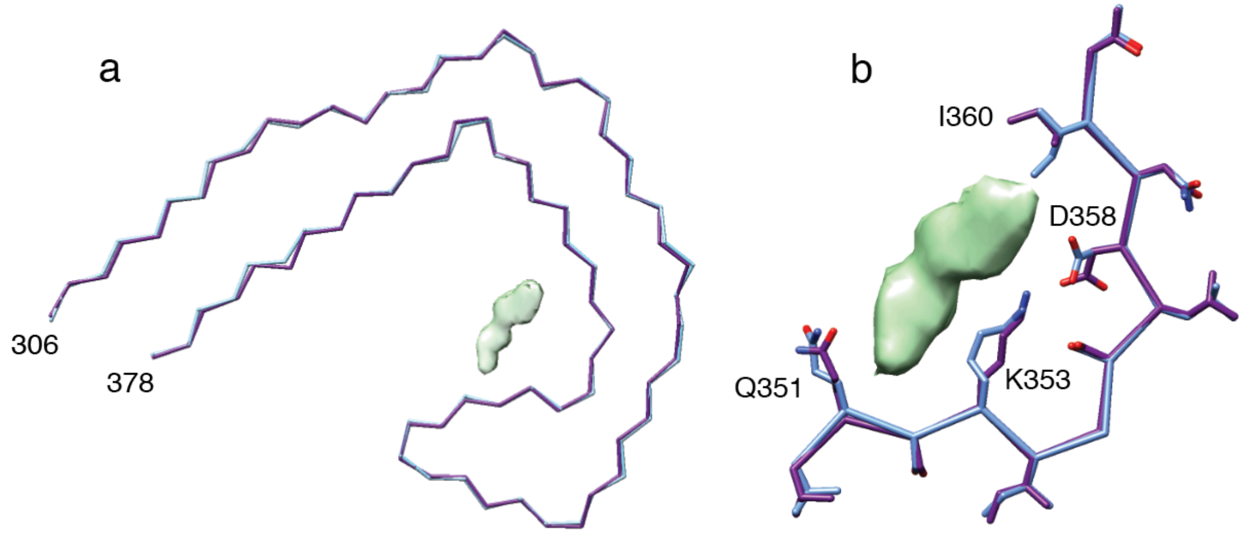

Supplementary Figure 5. Comparison of GTP-1 bound and apo tau PHFs.
a. Overlay of the backbone structures of PHFs with (purple) and without (blue) GTP-1 bound. **b.** Overlay of the residues in the GTP-1 binding pocket with (purple) and without (blue) GTP-1 bound, showing subtle sidechain rearrangements.

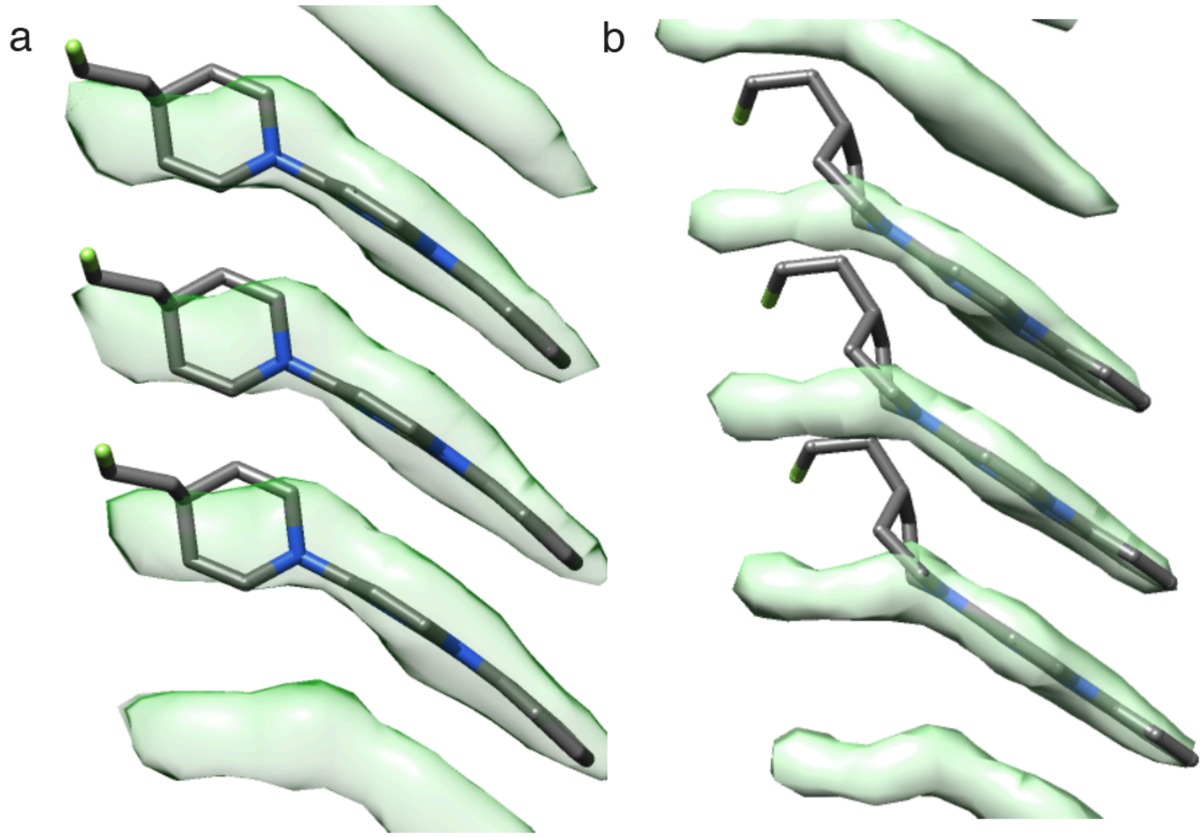

Supplementary Figure 6. Map+model of GTP-1 comparing Phenix and DFT optimization modeling approaches.

GTP-1 monomer conformations generated by (**a**) PHENIX eLBOW and (**b**) DFT optimization of a monomer fit into the ligand density. The distance of closest approach between stacked molecules is 2.3 Å for the eLBOW conformation and 1.7 Å for the DFT conformation.

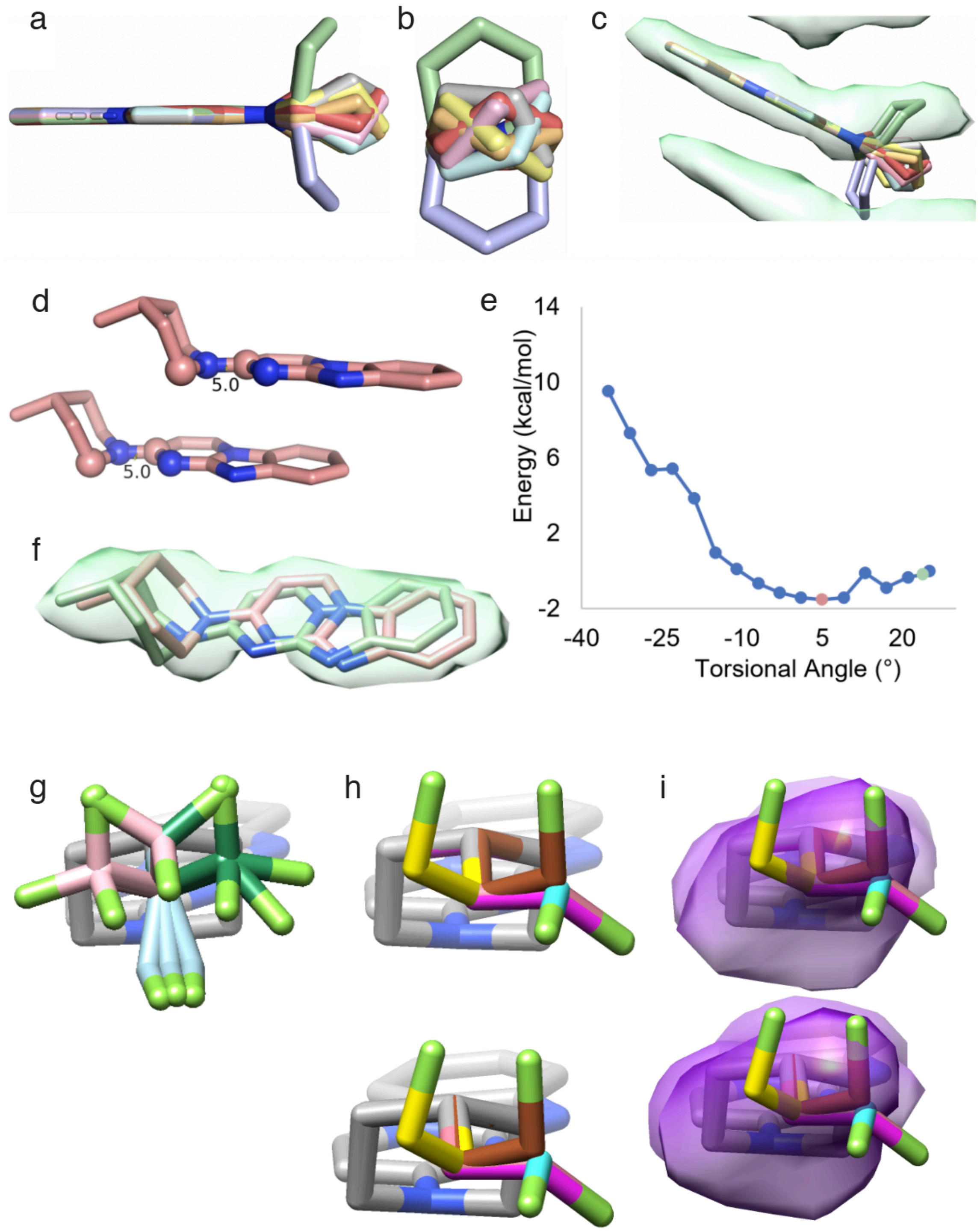

Supplementary Figure 7. Conformational search of the flexible nonaromatic region of GTP-1.

(**a**–**c**) Conformational search of the piperidine ring. The possible conformers are related by one mirror plane in the plane of the tricycle and a pseudo-mirror plane that is orthogonal to the plane of the tricycle and lies along the C–N bond connecting the tricycle to the piperidine ring. **a.** Edge-on view of centroids resulting from piperidine ring search. Centroids generated with a maximum

atom distance of 0.5 Å. **b.** End-on view of piperidine ring centroids. **c.** Comparison of the centroids placed into the density, which clarifies that the light green centroid is the correct ring conformation. (**d**–**f**) Torsional search of the piperidine ring. The potential energy surface is very soft except for where clash occurs with the dimer. The energy minimum presumably arises from better donation of the piperidine nitrogen into the aromatic tricycle. **d.** Example of one output from the constrained DFT optimizations. The torsional angle defined by the atoms indicated as spheres were constrained. Also, the translational distance between all of the atoms in the piperidine ring was constrained across the dimer. **e.** A graph of the energy landscape for the torsional angle. The green dot was the Maestro output (23.8°), and the coral dot (5°) was the minimum energy angle found. **f.** The starting Maestro conformation (green) and the torsion-optimized conformation (coral) in the cryo-EM density showing the improved fit upon torsional optimization. (**g**–**i**) Conformational search of the fluoroethyl tail. In the absence of the protein and the small molecule stack, the fluoroethyl conformers occupy essentially a three-fold symmetric well with a pseudo-mirror plane that is along the C–N bond connecting the tricycle to the piperidine ring and that is orthogonal to the tricycle. **g.** All of the outputs from the search in Maestro using 0.1 Å atom maximum distance. The conformers in light blue clashed with their dimeric partner, the conformers in pink clashed with the protein, and the conformers in green were taken forward for constrained optimization as dimers. **h.** The dimers that resulted from constrained optimization of the Maestro outputs as dimers using a translational constraint on the piperidine ring and fluoroethyl tail atoms. **i.** Dimer outputs compared to the density. The bright brown monomer was used as the input for the final Phenix refinement.

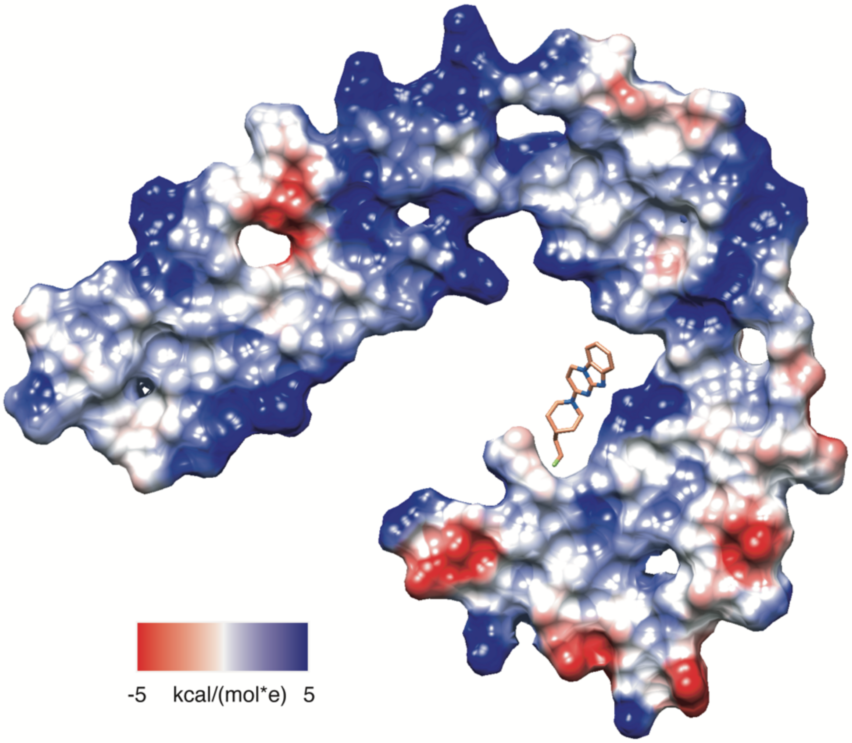

Supplementary Figure 8. Solvent accessible surface of an AD PHF with GTP-1 modeled. GTP-1 binds in a cleft in the AD PHF that shows strong geometric and electrostatic complementarity to the small molecule. To quantitatively understand this complementarity, we also performed this calculation on a per-residue basis (for all residues that made a heavy atom contact of ≤ 4.0 Å; Supplementary Table 2). The two strongest interactions are polar: the bifurcated hydrogen bond between the lysine side chain and 2 nitrogens on the aromatic ring of GTP-1 (~33%) and the non-canonical hydrogen bond between the oxygen of the Gln351 side chain and the beta-carbon of the fluoroethyl tail of GTP-1 (~25%). Together however, these interactions provide only ~58% of the binding energy, leaving the other ~42% to hydrophobic interactions which are driven by the geometry of the pocket and the shape of GTP-1. The concavity of the cleft makes close hydrophobic interactions at both ends of GTP-1 possible, and while there are other areas of concavity in the protofilament and between the paired filaments, this pocket is the only one which complements the electrostatics of GTP-1.

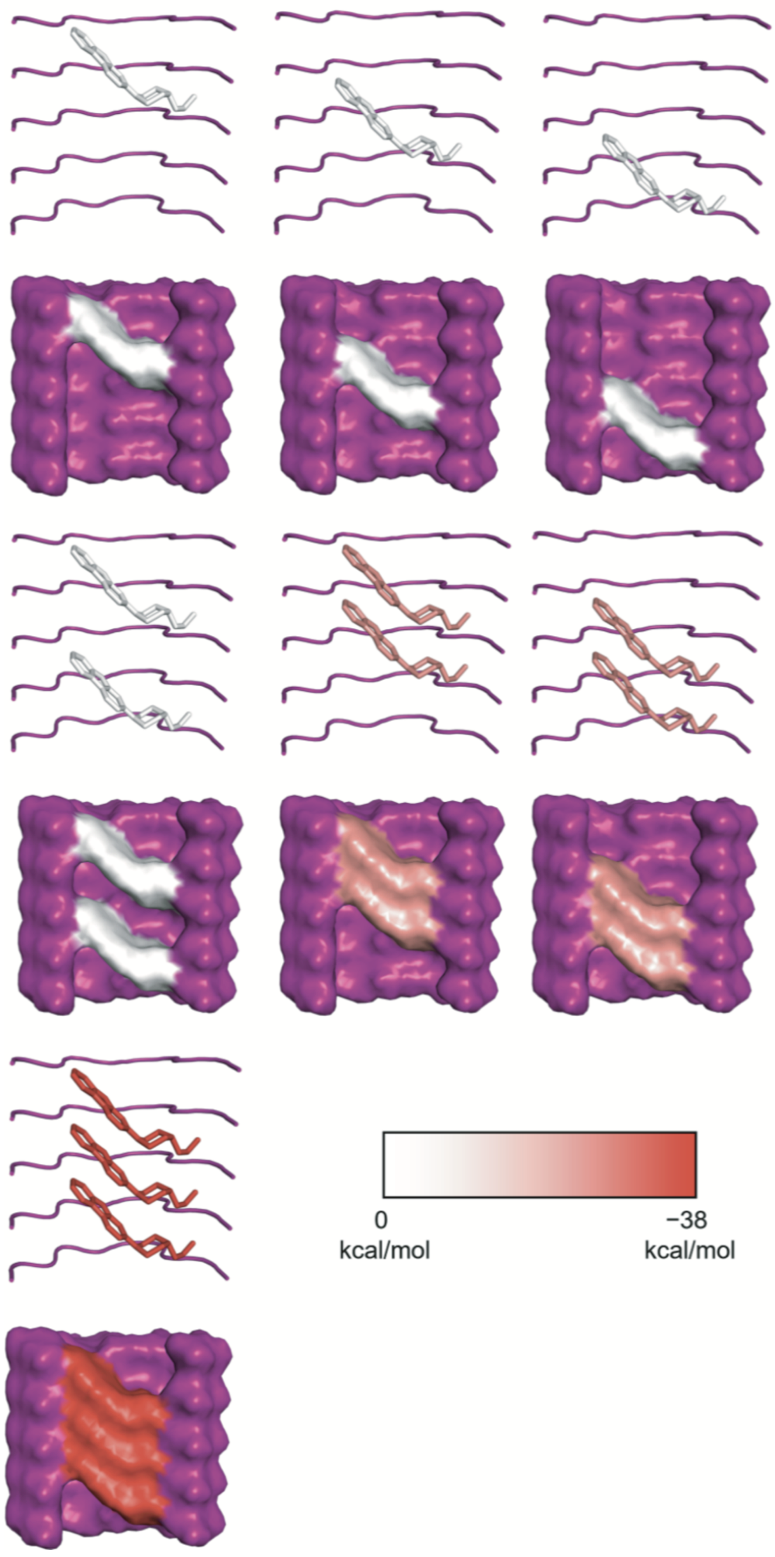

Supplementary Figure 9. Energetics of GTP-1 binding to the amyloid filament.
The ligands in the binding pocket (purple) with their energy represented along a color bar from 0 kcal/mol (blue) to −38 kcal/mol (red), with more negative energies being more favorable.

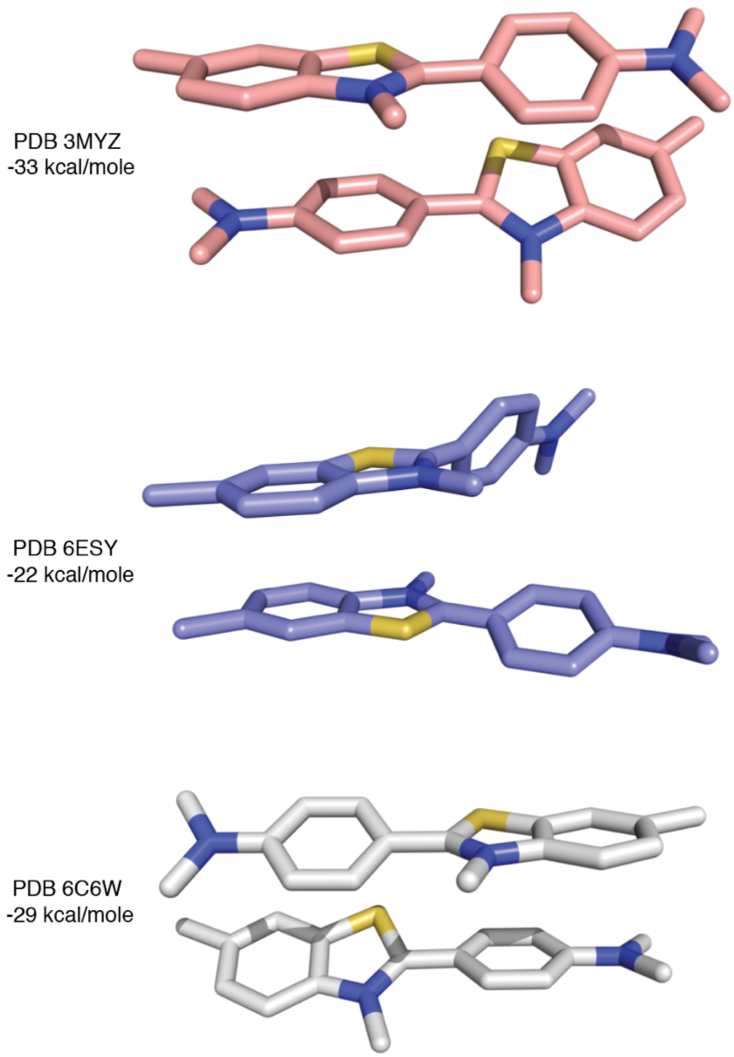

Supplementary Figure 10. Dimerization energy of ThT dimers found in crystal structures. Dimer conformations of ThT found in crystal structures of soluble proteins, and the ThT dimerization energy calculated by HFLD.

Supplementary Table 1. Cryo-EM data collection and refinement statistics.

**Data collection and processing**	**PHF + GTP-1**
Microscope and camera	Titan Krios, K3
Magnification	105,000
Voltage (kV)	300
Electron exposure ($e^-/Å^2$)	46
Dose rate (e^-/physical pixel/sec)	16
Exposure per frame (sec)	0.024
Defocus range (µm)	−0.8 to −1.8
Physical pixel size (Å)	0.834
Movies collected	15,160
Box size (pixels)	288
Interbox distance (Å)	28
Initial segments extracted	380,428
Final segments	30,199
Resolution (Å)	2.7
B-factor ($Å^2$)	−42.9
Helical rise (Å)	2.37
Helical twist (°)	179.45
**Refinement**	**Tau PHF: GTP-1**
**Model Composition**	
Non-hydrogen atoms	5,810
Protein residues	730
Ligands	10
**R.M.S. Deviations**	
Bond Lengths (Å)	0.004
Bond angles (°)	0.988
**Validation**	
Molprobity score	2.30
Clashscore	6.91
Rotamer outliers (%)	3.1
Cβ outliers (%)	4.35
**Ramachandran Plot**	
Favored (%)	90.14
Allowed (%)	9.86
Outliers (%)	0
PDB accession code	8FUG
EMDB accession code	EMD-29458

Supplementary Table 2. Single point DFT calculations of GTP-1 interactions with tau.

Residue	Strand	Interaction Energy (kcal/mol)
Asp-356	1	0.9
Ile-360	1	3.0
Gln-351	2	1.1
Ser-352	2	1.3
Lys-353	2	5.0
Gln-351	3	3.9

Supplementary Table 3. Single point DFT calculations and surface area calculations of GTP-1 with tau.

Species	Electronic Energy (Hartrees)	$\Delta E_{binding}$ (kcal/mol)	$\Delta\Delta E_{binding}$ (kcal/mol)	Surface Area (Å^2)	ΔSurface Area (Å^2)
Apo	-18,371.533	N/A	N/A	3,743.6	N/A
Optimized GTP-1	-977.489	N/A	N/A	N/A	N/A
Modeled GTP-1	-977.483	N/A	N/A	531.3	N/A
2 Modeled GTP-1	-1,954.997	N/A	N/A	768.2	N/A
GTP-1 (top)	-19,349.073	−35.5	−0.3	3,472.4	−1.2
GTP-1 (middle)	-19,349.074	−35.8	−0.6	3,473.3	−0.3
GTP-1 (bottom)	-19,349.073	−35.2	0	3,742.7	−0.9
2 GTP-1 (top)	-20,326.644	−90.6	−19.6	3,658.5	−85.1
2 GTP-1 (bottom)	-20,326.644	−90.3	−19.3	3,658.0	−85.6
2 GTP-1 (gap)	-20,326.613	−71.2	−0.2	3,741.4	−2.2
3 GTP-1	-21,304.215	−145.6	−38.8	3,573.3	−170.3

Supplementary References

1 Sanders, D. W. *et al.* Distinct Tau Prion Strains Propagate in Cells and Mice and Define Different Tauopathies. *Neuron* **82**, 1271-1288 (2014).

2 Degorce, F. *et al.* HTRF: A technology tailored for drug discovery - a review of theoretical aspects and recent applications. *Curr Chem Genomics* **3**, 22-32 (2009).

3 Fitzpatrick, A. W. P. *et al.* Cryo-EM structures of tau filaments from Alzheimer's disease. *Nature* **547**, 185–190 (2017).
