# Supplementary material for: Stacked binding of a PET ligand to Alzheimer’s tau paired helical filaments
Source: Nat Commun. 2023 May 26;14:3048. doi: 10.1038/s41467-023-38537-y (PMC10220082; doi:10.1038/s41467-023-38537-y)
Supplement: Supplementary file 3 — Description of Additional Supplementary Files [file 41467_2023_38537_MOESM3_ESM.pdf]

<u>Description of Additional Supplementary Files</u>

Supplementary Dataset 1. Conformers generated during GTP-1 modeling.
PDB files of all outputs generated along the GTP-1 modeling path described in Methods.

Supplementary Dataset 2. Coordinates from molecular dynamics simulations.
Initial and final coordinates of two 100 ns MD simulations of the PHF:GTP-1 complex. As each PHF contains two protofilaments, the simulations comprise four tau:GTP-1 complexes.
