# Supplementary material for: Stacked binding of a PET ligand to Alzheimer’s tau paired helical filaments
Source: Nat Commun. 2023 May 26;14:3048. doi: 10.1038/s41467-023-38537-y (PMC10220082; doi:10.1038/s41467-023-38537-y)
Supplement: Supplementary file 6 — Reporting Summary [file 41467_2023_38537_MOESM6_ESM.pdf]

nature portfolio

Corresponding author(s): Daniel Southworth
Last updated by author(s): Mar 30, 2023

Reporting Summary

Nature Portfolio wishes to improve the reproducibility of the work that we publish. This form provides structure for consistency and transparency in reporting. For further information on Nature Portfolio policies, see our Editorial Policies and the Editorial Policy Checklist.

Statistics

For all statistical analyses, confirm that the following items are present in the figure legend, table legend, main text, or Methods section.

n/a	Confirmed	
☐	☒	The exact sample size (*n*) for each experimental group/condition, given as a discrete number and unit of measurement
☐	☒	A statement on whether measurements were taken from distinct samples or whether the same sample was measured repeatedly
☒	☐	The statistical test(s) used AND whether they are one- or two-sided<br>*Only common tests should be described solely by name; describe more complex techniques in the Methods section.*
☒	☐	A description of all covariates tested
☒	☐	A description of any assumptions or corrections, such as tests of normality and adjustment for multiple comparisons
☐	☒	A full description of the statistical parameters including central tendency (e.g. means) or other basic estimates (e.g. regression coefficient) AND variation (e.g. standard deviation) or associated estimates of uncertainty (e.g. confidence intervals)
☒	☐	For null hypothesis testing, the test statistic (e.g. *F*, *t*, *r*) with confidence intervals, effect sizes, degrees of freedom and *P* value noted<br>*Give P values as exact values whenever suitable.*
☒	☐	For Bayesian analysis, information on the choice of priors and Markov chain Monte Carlo settings
☒	☐	For hierarchical and complex designs, identification of the appropriate level for tests and full reporting of outcomes
☒	☐	Estimates of effect sizes (e.g. Cohen's *d*, Pearson's *r*), indicating how they were calculated

Our web collection on statistics for biologists contains articles on many of the points above.

Software and code

Policy information about availability of computer code

Data collection	Serial EM 3.8; Scipion 3.0.12
Data analysis	RELION3.1; Chimera 1.13.1; COOT v0.8.9.2; Phenix 1.20.1; Maestro; ORCA; AMBER18; Gaussian09; MotionCorr2 1.5.0

For manuscripts utilizing custom algorithms or software that are central to the research but not yet described in published literature, software must be made available to editors and reviewers. We strongly encourage code deposition in a community repository (e.g. GitHub). See the Nature Portfolio guidelines for submitting code & software for further information.

Data

Policy information about availability of data

All manuscripts must include a data availability statement. This statement should provide the following information, where applicable:

- Accession codes, unique identifiers, or web links for publicly available datasets
- A description of any restrictions on data availability
- For clinical datasets or third party data, please ensure that the statement adheres to our policy

Cryo-EM maps and atomic coordinates have been deposited in the EMDB and PDB with accession codes: EMD-29458 [https://www.ebi.ac.uk/emdb/EMD-29458] and PDB 8FUG [http://doi.org/10.2210/pdb8FUG/pdb]. Any other relevant data are available from the corresponding author upon request.

Human research participants

Policy information about studies involving human research participants and Sex and Gender in Research.

Reporting on sex and gender	The tissue used in this study was from a male patient. Sex was not considered in this study, as the structure of Alzheimer's disease filaments does not vary by sex.
Population characteristics	Tissue from an individual with clinically diagnosed and neuropathologically confirmed Alzheimer's disease was used. The individual was an 88 year old man.
Recruitment	The sample was selected based on brain tissue availability and post-mortem neuropathological examination.
Ethics oversight	Alzheimer's disease tissue was obtained from the UCSF Neurodegenerative Disease Brain Bank (NDBB). These experiments were approved by the Neurodegenerative Disease Brain Bank Institutional Review Board 11-05588 and the UCSF Memory and Aging Center Autopsy Program Institutional Review Board 12-10512. This research was conducted in accordance with the principles of the Declaration of Helsinki. Informed consent was obtained from the patient's next of kin, including for identifying data to be shared with researchers using tissue obtained from the UCSF NDBB.

Note that full information on the approval of the study protocol must also be provided in the manuscript.

Field-specific reporting

Please select the one below that is the best fit for your research. If you are not sure, read the appropriate sections before making your selection.

☒ Life sciences ☐ Behavioural & social sciences ☐ Ecological, evolutionary & environmental sciences

For a reference copy of the document with all sections, see nature.com/documents/nr-reporting-summary-flat.pdf

Life sciences study design

All studies must disclose on these points even when the disclosure is negative.

Sample size	A sample size of 1 was used, as frozen patient tissue is quite rare and difficult to acquire. However, all Alzheimer's disease tau PHF structures from patient tissue characterized so far have the same fold, leading us to believe that GTP-1 binding would occur in the same manner with tissue from other patients with Alzheimer's disease.
Data exclusions	Pre-established common image classification procedures (Scheres, J. Struc. Biol. 180: 519-530, (2012)) were used to select particle images with the highest resolution in the image reconstruction process. Details of the number of images are given in Supplementary Table 1.
Replication	This structure represents an average of more than 380,000 images, and thus we believe our conclusions to be quite robust.
Randomization	This is not relevant to our study, there was only 1 sample.
Blinding	This is not relevant to our study, there was no group allocation.

Reporting for specific materials, systems and methods

We require information from authors about some types of materials, experimental systems and methods used in many studies. Here, indicate whether each material, system or method listed is relevant to your study. If you are not sure if a list item applies to your research, read the appropriate section before selecting a response.

Materials & experimental systems

n/a	Involved in the study	
☒	☐	Antibodies
☐	☒	Eukaryotic cell lines
☒	☐	Palaeontology and archaeology
☒	☐	Animals and other organisms
☒	☐	Clinical data
☒	☐	Dual use research of concern

Methods

n/a	Involved in the study	
☒	☐	ChIP-seq
☒	☐	Flow cytometry
☒	☐	MRI-based neuroimaging

Eukaryotic cell lines

Policy information about cell lines and Sex and Gender in Research

Cell line source(s)	All human tau-overexpressing cell lines were generated in HEK-293 cells purchased from ATCC (CRL-3216).
Authentication	None of the cell lines used were authenticated.
Mycoplasma contamination	Cell lines were not tested for mycoplasma contamination.
Commonly misidentified lines (See ICLAC register)	No commonly misidentified cell lines were used.
